# Aromatase inhibitors and fracture prevention – do 2017 guidelines work in real world?

**DOI:** 10.1038/s44276-024-00059-5

**Published:** 2024-05-01

**Authors:** Anem Mirza, Zeyar Win Naing, Parisa Khonsari, Haseeb Khan, Ali K. Abbas, Muhammad K. Nisar

**Affiliations:** 1 University College Hospital, 235 Euston Road., London, NW1 2BU UK; 2 Luton & Dunstable University Hospital, Lewsey Road, Luton, LU4 0DZ UK

## Abstract

**Objectives:**

Aromatase inhibitor induced bone loss (AIBL) is a recognised adverse event with resultant increase in fracture risk. We aimed to determine the real-world impact of the 2017 consensus guidelines on AIBL and see if it is effective in fracture prevention.

**Methods:**

Over a 7-year study period, 1001 women prescribed AI were split in two groups. First group were offered bone active treatment based on NOS 2008 guidelines whereas the second group followed the 2017 consensus guidelines.

**Results:**

1001 women were included.

First group: 361 women had a baseline DEXA with 143 (40%) women who had a normal DEXA, 174 (48%) had osteopenia and 44 (12%) had osteoporosis. Of the women with osteopenia, 44 (25%) women were offered treatment, and 22 (13%) women had a fracture. Second group: 640 women had a baseline DEXA with 216 (33%) women with a normal result, 322(50%) had osteopenia and 107 (17%) had osteoporosis. Of the women with osteopenia, 127 (39%) women were offered treatment, and 8 (2.5%) women had a fracture.

**Conclusions:**

Our study provides real world evidence of the success of 2017 consensus statement in lowering fracture risk. A significant reduction in fractures pre (13%) and post guidelines change (2.5%) was demonstrated (absolute risk reduction of 10.5%) which has implications for healthcare systems worldwide as we have demonstrated this approach can reduce morbidity.

**Lay summary:**

Breast cancer is the most common cancer in women with over two million women diagnosed with it annually. Early diagnosis and treatment with hormonal therapies have helped reduce mortality. Aromatase inhibitors (AIs) are the main drugs in this class and have demonstrated improved survival. However, whilst conveying major benefits, AIs reduce oestrogen levels leading to significant bone loss and increasing fracture risk. Several protocols have been recommended to address this concern. We compared the two guidelines published by National Osteoporosis Society UK in 2008 and consensus statement recommended by seven breast cancer and bone health groups in 2017 to see which work better in preventing fractures in women prescribed AIs for breast cancer. Our study shows that the 2017 guidelines are better at preventing fractures in the real world. Hence, we suggest that these should be adopted by specialists treating breast cancer which can help women avoid fractures and improve long term health.

## Introduction

The incidence of breast cancer has been steadily rising and represents the most common cancer in women. Over two million women are diagnosed with breast cancer worldwide every year. This accounts for over 30% of female neoplasms. Lifetime risk of developing breast cancer in women has been shown to be nearly one in eight with the 5-year overall survival estimated to be 80% or higher in high-income countries [1].

Early diagnosis and the employment of efficacious personalised treatments such as endocrine therapies have conferred significant mortality benefit [2]. Considering 80% of breast cancers are oestrogen receptor positive in women aged over fifty years, this makes drugs such as aromatase inhibitors (AIs) a highly attractive option. Both steroidal (exemestane) and non-steroidal (anastrozole and letrozole) AIs achieve over 98% aromatase inhibition in post-menopausal women with 80–90% resultant drop in peripheral oestradiol level. This is crucial in delivering improved survival for these patients [3].

Whilst conveying major benefits, the oestrogen deficiency induced by AIs leads to a significant increase in bone resorption and accelerated bone loss, especially at the trabecular bones [4]. The effects of adjuvant therapy on bone mineral density (BMD) have been quantified by several studies that have reported an annual bone loss in healthy postmenopausal women of 1–2% per year, while AIs therapy alone causes 2–3% BMD loss per year, greater during the first year, and progressively lower in the following years [5]. This translates into higher fracture risk with up to one in five women prescribed AIs reported to sustain a clinical fracture and nearly a third with incident vertebral fracture on morphometric analysis [6].

To address the AIs associated bone health concerns, a consensus statement of seven international bone and cancer societies was published in 2017 proposing an algorithm based on clinical risk factors and different BMD threshold for bone active therapeutic intervention [7]. Our study aims to determine the real-world impact of the 2017 consensus guidelines on AIs induced bone loss and whether bone sparing therapy utilising proposed risk stratification model is effective in fracture prevention compared to the 2008 UK recommendations [8].

## Methods

We undertook a retrospective study of patients prescribed AI for breast cancer over a seven-year period at our university teaching hospital. All the data was recorded electronically with full access to demographics, disease parameters, investigations, and drug management. Dual energy X-ray absorptiometry (DXA) scans performed prior to initiation of AI were compared with subsequent imaging over a mean follow up of 3 years. Outcome data for cancer and all fractures was collected. Descriptive statistics were employed to investigate significant relationships amongst the variables of interest. The project was approved on Jan 7, 2022 (approval number 13/2021-22/Medicine/Rheumatology).

Over a 7-year study period, 1001 women were prescribed AI at our university teaching hospital by rheumatologists working in the rheumatology department. The new guidelines were adopted in July 2017. We split the participants in two groups: 361 (36%) women had commenced their AI prior to the adoption of guidelines (Group One) and 640 (64%) were in the post implementation group (Group Two).

First group were offered bone active treatment based on National Osteoporosis Society (NOS) 2008 guidelines whereas the second group followed the 2017 consensus guidelines. The inhouse protocols for treatment were based on these guidelines. Women with osteoporosis were all offered treatment, however the difference in guideline is pertinent to osteopenia and we compared the results of that group. All patients with osteopenia or osteoporosis were advised to take calcium and vitamin D.

Patients were followed for bone health by having a repeat DXA scan. Fractures whilst on AIs were either diagnosed by imaging or a verbal report was received from a patient that they had sustained a fracture.

## Results

1001 women were included. Mean age was 64 years (range 29–93). 929 (93%) were Caucasian, 57 (6%) were Asian and 15 (1%) were Afro-Caribbean. 723 women (72%) had invasive ductal carcinoma and 863 women (86%) were postmenopausal. At diagnosis, 428 women (43%) had node positive disease and 35 women (4%) had metastases. 91 women (9%) had sustained fractures prior to their cancer diagnosis [Table 1].

**Table 1:** Demographics of the two cohorts.

	**Group one**	**Group two**
*n*	361	640
Mean age (years)	64	64
Ethnicity		
Afro-caribbean	10	5
Asian	21	36
Cacausian	330	599
Menopause status		
Pre-menopausal	24 (7%)	46 (7%)
Peri menopausal	21 (6%)	47 (7%)
Post menopausal	316 (87%)	547 (86%)
Invasive ductal carcinoma	204 (57%)	519 (81%)
Cancer grade at diagnosis		
1	62 (17%)	101 (16%)
2	202 (56%)	369 (58%)
3	97 (27%)	170 (26%)
Lymph node involvement at diagnosis		
Yes	177 (49%)	251 (39%)
No	184 (51%)	389 (61%)
Metastases at diagnosis		
Yes	8 (2%)	27 (4%)
No	353 (98%)	613 (96%)
Existing diagnosis of condition associated with secondary osteoporosis		
Chronic liver disease	0	1
Chronic malabsorption	0	1
Chronic malnutrition	0	0
Hypogonadism	0	0
Osteogenesis imperfecta	0	0
Premature menopause (< 45 years)	13	10
Type one diabetes	0	5
Untreated longstanding hyperthyroidism	1	0
Usage of steroids	9	14
Mean duration of follow up (years)	4	3

1001 women were included. Mean age was 64 years (range 29–93). 929 (93%) were Caucasian, 57 (6%) were Asian and 15 (1%) were Afro-Caribbean. 723 women (72%) had invasive ductal carcinoma and 863 women (86%) were postmenopausal. At diagnosis, 428 women (43%) had node positive disease and 35 women (4%) had metastases, 23 women (2%) had experienced premature menopause.

276 women (83%) were offered oral bisphosphonates based on DEXA result, with 58 (17%) offered parenteral therapy.

### First group

361 women had a baseline DEXA with a mean femoral neck left (FN left). BMD of 0.888 g/cm2 (range 0.552–1.222) [Table 2].

**Table 2:** Baseline DXA measurements of the two groups at diagnosis.

	Group one	Group two
*n*	361	640
Lumbar spine (L1-4) BMD (g/cm2)		
Mean	1.129	1.131
Median	1.121	1.116
Minimum	0.714	0.118
Maximum	1.806	2.066
Hip Left BMD (g/cm2)		
Mean	0.931	0.924
Median	0.928	0.914
Minimum	0.550	0.451
Maximum	1.262	1.430
Hip Right BMD (g/cm2)		
Mean	0.935	0.929
Median	0.938	0.916
Minimum	0.544	0.017
Maximum	1.267	1.609
Femoral Neck Left BMD (g/cm2)		
Mean	0.888	0.888
Median	0.886	0.870
Minimum	0.552	0.512
Maximum	1.222	1.390
Femoral Neck Right BMD (g/cm2)		
Mean	0.894	0.893
Median	0.886	0.878
Minimum	0.505	0.584
Maximum	1.251	1.607

At diagnosis of breast cancer, all women had a baseline DXA. In Group One, the mean femoral neck (FN) left BMD was 0.888, for Group Two it was also 0.888.

Note: *BMD* bone mineral density.

143 (40%) women had a normal DXA, 174 (48%) had osteopenia and 44 (12%) had osteoporosis.

**Fig. 1:**
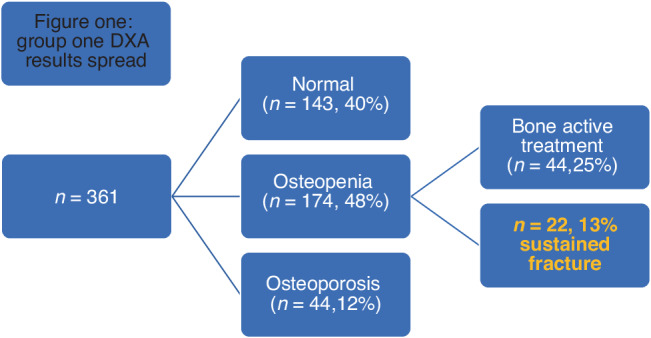
Group one DXA results spread.

Of the women with osteopenia (*n* = 174), 22 (12.6%) suffered fractures [Fig. 1]. 44 (25%) women were offered treatment based on 2009 NOS guidelines and 33 women had a repeat DXA after a mean of 4 years. In the treatment group, FN left mean BMD remained relatively unchanged from 0.814 g/cm2 to 0.812 g/cm2 at the repeat DEXA (*p* = 0.94) [Table 3]. 7/44 (16%) had fractures with no prior history of fractures with a median time to fracture of 1.5 years (range of 1–7 years). One lady was already prescribed bisphosphonates.

**Table 3:** Baseline DXA scores of individuals found to have osteopenia, and DXA scores at follow up for those who were offered bone active treatment.

	Group one	Follow up DEXA scan (Mean follow up time of 4 years)	Group two	Follow up DXA scan (Mean follow up time of 3 years)
*n*	44	33	127	56
Lumbar Spine (L1-4) BMD (g/cm2)				
Mean	1.050	1.039	1.081	1.032
Median	1.041	1.054	1.119	1.071
Minimum	0.887	0.827	0.877	0.843
Maximum	1.375	1.232	1.542	1.287
Hip Left BMD (g/cm2)				
Mean	0.853	0.853	0.867	0.872
Median	0.841	0.838	0.921	0.898
Minimum	0.718	0.723	0.690	0.699
Maximum	1.025	1.300	1.123	1.093
Hip Right BMD (g/cm2)				
Mean	0.846	0.840	0.872	0.881
Median	0.839	0.827	0.926	0.900
Minimum	0.741	0.729	0.705	0.739
Maximum	1.045	1.000	1.192	1.173
Femoral Neck Left BMD (g/cm2)				
Mean	0.814	0.812	0.822	0.830
Median	0.818	0.818	0.882	0.851
Minimum	0.698	0.689	0.690	0.651
Maximum	1.032	0.943	1.099	1.070
Femoral Neck Right BMD (g/cm2)				
Mean	0.807	0.831	0.824	0.841
Median	0.800	0.822	0.887	0.860
Minimum	0.708	0.699	0.680	0.674
Maximum	1.080	1.300	1.084	1.048

Of those women with osteopenia who were offered bone active treatment in Group One, FN left mean BMD remained relatively unchanged from 0.814 g/cm2 to 0.812 g/cm2 at the repeat DEXA. In Group Two, FN left mean BMD remained relatively unchanged from 0.822 g/cm2 to 0.829 g/cm2 at the repeat DEXA.

Note: *BMD* bone mineral density.

**Fig. 2:**
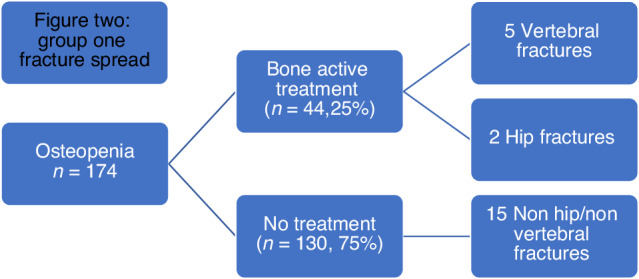
Group one fracture spread.

130/174 (75%) did not qualify for bone active treatment. 15/130 (11.5%) suffered fractures during follow up [Fig. 2].

### Second group

640 women had a baseline DEXA with a mean FN left BMD of 0.888 g/cm2 (range 0.512–1.390) [Table 2].

216 (33%) women had normal DXA, 322 (50%) had osteopenia and 107 (17%) had osteoporosis.

**Fig. 3:**
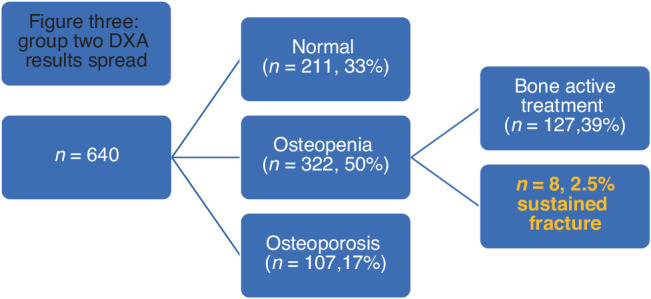
Group two DXA results spread.

Of the 322 women with osteopenia, 8 (2.5%) women had a fracture [Fig. 3].

127/322 (39%) women were offered treatment, and 56 women had a repeat DXA after a mean of 3 years. In the treatment group, FN left mean BMD remained relatively unchanged from 0.822 g/cm2 to 0.829 g/cm2 at the repeat DEXA (*p* = 0.6169) [Table 3]. 3/127 (2.3%) suffered fractures with a median time to fracture of 2 years (range 1–9 years).

**Fig. 4:**
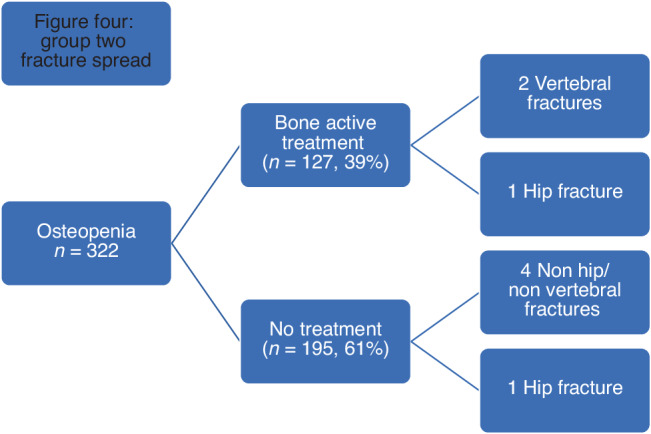
Group two fracture spread.

195/322 (61%) did not qualify for bone active treatment. 5/195 (2.5%) had a fracture during the follow up [Fig. 4].

## Discussion

Our study provides real world evidence of the success of 2017 consensus statement in lowering fracture risk. Though there has been data for positive impact on BMD decline with this approach, evidence for fracture prevention has been limited. This study showcases the success of lowering bone active therapy threshold employing alternative risk modelling strategy for women with breast cancer commenced on AI. A significant reduction in fractures pre (12.5%) and post guidelines change (2.5%) was demonstrated (absolute risk reduction of 10%) which has implications for healthcare systems worldwide as we have demonstrated this approach can reduce morbidity.

Poor bone health risk is well established in women with breast cancer owing to higher bone remodelling secondary to advanced age, hypovitaminosis D and employment of corticosteroids in chemotherapeutic regimens [9]. AIs are reported to nearly double this risk when annual bone loss rates are compared to healthy postmenopausal women [6]. The concern is even higher in premenopausal women prescribed endocrine therapy particularly those with premature ovarian failure due to chemotherapy [10].

Irrespective of the degree of BMD loss, AIs have been shown to confer higher fracture risk compared to placebo or Tamoxifen. The risk seems to be similar for all the approved agents [11]. Furthermore, the longer the exposure to AIs, the higher the risk of fractures. This is important as most guidelines recommend 5–10 years of AI treatment to improve cancer outcomes [12]. As it is mainly trabecular bone loss seen with AIs, vertebral fractures are the predominant manifestation with up to a third of women affected in a recent study [13].

Therefore, it’s imperative that fracture risk assessment tools consider the significant risk conferred by AIs especially when the probability of fracture is partly independent of BMD. This is pivotal in women with osteopenia as those with osteoporosis will be offered fracture prevention therapy anyway. Fracture risk assessment tool (FRAX) has been shown to work reasonably well in a large registry-based cohort study however the threshold to commence bone active therapy is subject to discussion captured in the variability of the guidelines [14].

In the UK, most centres follow 2008 Consensus position statement from a UK Expert Group which recommended bisphosphonates for postmenopausal women younger than 75 years with T-scores <−2.0 or with bone loss ⩾ 4% per year in pre-existing osteopenia (T scores between −1.1 to −2.4). At our centre, this translated to only a quarter of women with osteopenia qualifying for bisphosphonates leading to over one in ten women fracturing during follow up [7].

In 2017, The Joint position statement of the International Osteoporosis Foundation, Cancer and Bone Society, European Calcified Tissue Society, International Expert Group for AIBL, European Society for Clinical and Economics Aspects of Osteoporosis, Osteoarthritis, and Musculoskeletal Diseases, International Menopause Society and International Society for Geriatric Oncology recommended starting anti-osteoporotic therapy when T-score is <−2.0 or with ⩾ 2 risk factors (including T-score <−1.5). When we switched to these guidelines, an extra 15% women qualified for bone active therapy thereby achieving absolute fracture reduction of 10% compared to the earlier cohort [8]. Our study confirms the superiority of the 2017 joint position statement which were reinforced by European Society of Medical Oncology (ESMO) [15].

All guidelines concur on the need to offer bone sparing therapy for women with osteoporosis however the differences exist in recommendations for osteopenia. Our study reaffirms that a significant number of fractures occur in women with osteopenia. The differential improvement in fractures observed is largely down to the extra 15% of women qualifying for antiresorptive therapy based on 2017 guidelines. This endorses the need for holistic risk assessment and lower threshold for intervention in women with low bone mass considering all risk factors.

There are several caveats to consider including monocentric, retrospective nature of the study with two unmatched cohorts in a diverse ethnic setting thus making generalisability of the findings difficult. Additionally, data was not collected on markers of bone turnover, vitamin D levels or administration of concomitant glucocorticosteroids or chemotherapy which may have been confounding factors. However, the strengths include a large cohort with a long follow up period, availability of comprehensive clinical data and ability to provide hard outcome of fracture rather than BMD data.

## Data Availability

Data available on request from the authors.

## References

[1] Loibl S, Poortmans P, Morrow M. Breast cancer. Lancet. 2021;397:1750–69. 33812473 10.1016/S0140-6736(20)32381-3

[2] Early Breast Cancer Trialists Collaborative Group (EBCTCG) Aromatase inhibitors versus tamoxifen in early breast cancer: patient-level meta-analysis of the randomised trials. Lancet. 2015;386:1341–52. 26211827 10.1016/S0140-6736(15)61074-1

[3] Chien AJ, Goss PE. Aromatase inhibitors and bone health in women with breast cancer. J Clin Oncol. 2006;24:5305–12. 17114665 10.1200/JCO.2006.07.5382

[4] Shapiro CL. Bone-modifying Agents (BMAs) in breast cancer. Clin Breast Cancer. 2021;21:e618–30. 34045175 10.1016/j.clbc.2021.04.009

[5] Shapiro CL, Van Poznak C, Lacchetti C, Kirshner J, Eastell R, Gagel R, et al. Management of osteoporosis in survivors of adult cancers with nonmetastatic disease: ASCO clinical practice guideline. J Clin Oncol. 2019;37:2916–46. 31532726 10.1200/JCO.19.01696

[6] Tseng OL, Spinelli JJ, Gnant M, Brandi ML, Reginster JY, Gotay CC, Ho WY, McBride ML, Dawes MG. Aromatase inhibitors are associated with a higher fracture risk than tamoxifen: a systematic review and meta-analysis. Ther Adv Musculoskelet Dis. 2018;10:71–90. 29619093 10.1177/1759720X18759291 PMC5871065

[7] Hadji P, Aapro MS, Body JJ, et al. Management of Aromatase Inhibitor-Associated Bone Loss (AIBL) in postmenopausal women with hormone sensitive breast cancer: joint position statement of the IOF, CABS, ECTS, IEG, ESCEO IMS, and SIOG. J Bone Oncol. 2017;7:1–12. 28413771 10.1016/j.jbo.2017.03.001 PMC5384888

[8] Reid DM, Doughty J, Eastell R, Gnant M, Brandi ML, Reginster JY, et al. Guidance for the management of breast cancer treatment-induced bone loss: a consensus position statement from a UK Expert Group. Cancer Treat Rev. 2008;34:S3–S18. 18515009 10.1016/j.ctrv.2008.03.007

[9] Kanis JA, Cooper C, Rizzoli R, Reginster JY. Executive summary of the European guidance for the diagnosis and management of osteoporosis in postmenopausal women. Calcif Tissue Int. 2019;104:235–8. 30796490 10.1007/s00223-018-00512-x PMC6422308

[10] Guise TA. Bone loss and fracture risk associated with cancer therapy. Oncologist. 2006;11:1121–31. 17110632 10.1634/theoncologist.11-10-1121

[11] Berruti A, Tucci M, Mosca A, Vana F, Ardine M, Dogliotti L, et al. Changes in bone mineral density after adjuvant aromatase inhibitors and fracture risk in breast cancer patients. J Clin Oncol. 2007;25:1455–6. 17416873 10.1200/JCO.2006.08.7080

[12] Cuzick J, Sestak I, Baum M, Buzdar A, Howell A, Doswett M, et al. Effect of anastrozole and tamoxifen as adjuvant treatment for early-stage breast cancer: 10-year analysis of the ATAC trial. Lancet Oncol. 2010;11:1135–41. 21087898 10.1016/S1470-2045(10)70257-6

[13] Pedersini R, Monteverdi S, Mazziotti G, Amoroso V, Roca E, Maffezzoni F, et al. Morphometric vertebral fractures in breast cancer patients treated with adjuvant aromatase inhibitor therapy: a cross-sectional study. Bone. 2017;97:147–52. 28104509 10.1016/j.bone.2017.01.013

[14] Leslie WD, Morin SN, Lix LM, Niraula S, McCloskey EV, Johansson H, et al. Performance of FRAX in women with breast cancer initiating aromatase inhibitor therapy: a registry-based cohort study. J Bone Miner Res. 2019;34:1428–35. 31069862 10.1002/jbmr.3726

[15] Coleman R, Hadji P, Body J, Santini D, Chow E, Terpos E, et al. Bone health in cancer: ESMO clinical practice guidelines. Ann Oncol. 2020;31:1650–63. 32801018 10.1016/j.annonc.2020.07.019

